# Nutrient dynamics in water and soil under conventional rice cultivation in the Vietnamese Mekong Delta

**DOI:** 10.12688/f1000research.73904.1

**Published:** 2021-11-11

**Authors:** Nguyen Vo Chau Ngan, Huynh Van Thao, Nguyen Dinh Giang Nam

**Affiliations:** 1 Department of Water Resources - College of Environment and Natural Resources, Can Tho University, Can Tho city, 900000, Vietnam; 2 Department of Environmental Science - College of Environment and Natural Resources, Can Tho University, Can Tho city, 900000, Vietnam

**Keywords:** nutrient availability, nutrient loss, surface water, subsurface water, soil, the Vietnamese Mekong Delta, water management

## Abstract

**Background** The evaluation of nutrient variability plays a crucial role in accessing soil potentials and practical intervention responses in rice production systems. Synthetic fertilizer applications and cultivation practices are considered key factors affecting nutrient dynamics and availability. Here, we assessed the nutrient dynamics in surface, subsurface water and soil under local water management and conventional rice cultivation practices in the Vietnamese Mekong Delta.

**Methods** We implemented a field experiment (200 m ^2^) in the 2018 wet season and the 2019 dry season in a triple rice-cropping field. Eight samples of surface water, subsurface water (30–45 cm), and topsoil (0–20 cm) were collected and analysed during the rice-growing seasons.

**Results** The results showed that N-NH _4_^+^, P-PO _4_^3−^ and total P peaks were achieved after fertilizing. Irrespective of seasons, the nutrient content in surface water was always greater than that of subsurface water ( *P* < 0.001), with the exception of N-NO _3_^−^, which was insignificant ( *P* > 0.05). When comparing the wet and dry seasons, nutrient concentrations exhibited minor differences ( *P* > 0.05). Under conventional rice cultivation, the effects of synthetic fertilizer topdressing on the total N, soil organic matter (SOM), and total P were negligible in the soil. Higher rates of N fertilizer application did not significantly increase soil N-NH _4_^+^, total N, yet larger P fertilizer amounts substantially enhanced soil total P ( *P* < 0.001).

**Conclusions** Under conventional rice cultivation, N-NH _4_^+^, P-PO _4_^3−^ and total P losses mainly occur through runoff rather than leaching. While N-NO _3_^−^ loss is similar in surface water and subsurface water. Notably, nutrient content in soil was high; whilst SOM was seen to be low-to-medium between seasons. Future work should consider the nutrient balance and dynamic simulation in the lowland soil of the Vietnamese Mekong Delta’s paddy fields.

## Introduction

The Mekong Delta (MD) is the biggest rice-producing region in Vietnam ( Clauss *et al.,* 2018), accounting for approximately 55% of total national rice ( *Oryza sativa L.*) outputs through intensive rice production systems ( Uno *et al.,* 2021). Here, double-and triple rice-cropping systems are the most commonly employed rice cultivation practices in the MD. Along with agronomic practices in intensive rice-based farming systems, a vast amount of fertilizer is typically applied to the paddy fields to obtain higher yields. For conventional rice cultivation in the MD’s paddy fields, the amount of fertilizer application in the wet season (WS) and dry season (DS) has been found to vary from 82–97 kg N ha ^−1^, 22.64–22.69 kg P ha ^−1^, and 29–32 kg K ha ^−1^ ( Stuart *et al.,* 2018). Common rice practices typically use fertilizers, water, and seeds well exceeding recommended rates ( Stuart *et al.,* 2018, Connor *et al.,* 2021). It is reported that the efficacy of N use by rice plants is generally relatively low, roughly 30–35%, while N loss to the environment is approximately 50% ( Zhu and Chen, 2002). Moreover, Irfan *et al.* (2020) and Schröder *et al.* (2011) revealed that P utilization efficacy varies 10–15%, whereas P loss to the environment ranges 9.7–12.4% and 0.3–0.5% for surface runoff and subsurface leaching, respectively ( Cho *et al*., 2011). In the rice field, fertilizer and water management regimes are key factors affecting transport, as well as the use efficacy of N and P ( Qi *et al.,* 2020). Yang *et al.* (2015) reported that different water and fertilizer management practices exported 13.1–31.7% N input to the environment, in which N loss through ammonia accounted for 69.6–83.5%. Furthermore, it has been noted that N loss from rice soils typically occurs through ammonia volatilization and nitrification-denitrification ( Shankar *et al.,* 2021), while loss of P was comparatively low due to the enrichment of Ca ^2+^, Fe ^3+^ and Al ^3+^ oxides which can adsorb P in several mineral forms ( Wang *et al.,* 2015; Scalenghe *et al.,* 2014). Several previous studies reported that nutrient migration was lost via surface water (SW) and sub-surface water (SbW) ( Peng *et al.,* 2011; Qi *et al.,* 2020; Schröder *et al.*, 2011; Wang and Huang, 2021). Thus, it has been suggested that higher amounts of fertilizer application under conventional rice cultivation and local water management regimes would largely result in increased nutrients in adjacent environments. To the best of our knowledge, quantitative variability of nutrients in SW, SbW and soil under conventional rice farming practices of the Vietnamese MD has not been previously studied. Therefore, this paper aims to explore the temporal-spatial dynamics of nutrients in the SW, SbW and soil of triple rice-cropping models both during the WS and DS under conventional local farming practices.

## Methods

### Study area

This study was conducted at a local farmer's field in Long Tuyen district, Can Tho city, Vietnam (9°59’19”N, 105°36’14”E), from 2018 to 2019. The field experiment was located in a lowland soil, which applied triple-cropping rice, an intensive rice production system. According to Dong *et al.* (2012), the soil area was classified as Thionic Glycesol ( International Union of Soil Sciences (IUSS) working group World Reference Base (WRB), 2015). The average weather data was annually recorded from 2015–2019 as follows: rainfall, 2,088.4 mm; humidity, 70.0–86.0%; sunshine, 2,467.4–2,695.4 hours ( DONRE, 2020). The initial soil physicochemical properties were as follows: bulk density, 0.98 g cm ^−3^; soil texture (sand, 1.9%, and clay, 66.4%); soil organic matter (SOM), 35.4 mg kg ^−1^.

### Experimental design

The size of the field experiment was 200 m ^2^ (20 m × 10 m). The field was enclosed by a soil bank with plastic sheet coverage. The plastic sheet was buried 20 cm under the ground’s surface to secure against leaks or intrusion into the nearby fields. We conducted the field experiment in two seasons, including summer–autumn 2018 (wet season) and winter–spring 2019 (dry season). In summer–autumn 2018, the field experiment incorporated rice straw into the soil using a hand tractor. The straw was residue from the previous rice-growing season (spring–summer season 2018). The field witnessed a 10-day fallow period before sowing. In the winter–spring 2019 season, the field underwent a three-month natural flooding season. Before sowing, the field was drained and harrowed by a hand tractor. The rice crop calendar of the two field experiments is show in Table 1. Table 1 shows the rice farming practices during the wet season 2018 (summer–autumn) and dry season (winter–spring). The main practices comprise the schedule of soil preparation (ploughing), sowing, irrigation, fertilization, drainage, and harvest.

**Table 1.** Rice crop calendar for the field experiment.

Practice	Wet season		Dry season	
	Date ^†^	DAS	Date ^†^	DAS
Ploughing	06/08/2018	−10	28/01/2019	−1
Sowing	16/08/2018	0	29/01/2019	0
Starting irrigation	22/08/2018	7	05/02/2019	8
Fertilization				
- 1 ^st^ topdressing	25/08/2018	10	07/02/2019	10
- 2 ^nd^ topdressing	04/09/2018	20	13/02/2019	16
- 3 ^rd^ topdressing	01/10/2018	47	23/02/2019	26
- 4 ^th^ topdressing	-	-	16/03/2019	47
Drainage	08/11/2018	85	23/04/2019	85
Harvest	18/11/2018	95	04/05/2019	95

† Date is formatted as dd/mm/yyyy; DAS = day after seeding.

### Rice cultivation and water management

Short-duration rice varieties of OM4900 and OM6976 cultivars for the WS and the DS were used, respectively. The varieties were obtained from Cuu Long Delta Rice Research Institution (CLRRI), Vietnam. The maturity of the two rice varieties varied from 95 to 100 days. The selection of varieties was based on common use and edaphological adaptation in this region. Pre-germinated seeds were sown at 150 kg ha ^−1^ under saturated soil by direct seeding. Water was supplied from a watershed near the field. Water management followed the locally typical water use practices. Water irrigation was started on the seventh day after seeding (DAS), re-irrigated 5–7 cm before fertilizing, always retaining a water level of 1–3 cm during heading and flowering, and openly drained ten days before harvesting. Multiple drainages, which are a simplified form of alternative wetting and drying (AWD) typically conducted in the VMD ( Uno *et al.,* 2021), were performed whenever water level naturally decreased 10 cm below the soil surface for the remaining cultivation period.

### Fertilizer application

We applied synthetic fertilizers according to locally conventional rice cultivation. In the WS, 129.5 kg N ha ^−1^ and 75 kg P _2_O _5_ ha ^−1^ were used in total. These topdressings were applied at intervals of 10, 20, and 47 DAS. The fertilizers were applied as follows: 55/42.5/32 kg N ha ^−1^, 25/25/25 kg P _2_O _5_ ha ^−1^. In the DS, chemical fertilizers were used with a total amount of 90 kg N ha ^−1^, 94 kg P _2_O _5_ ha ^−1^, and 70.5 kg K _2_O ha ^−1^. Fertilization was split into four intervals on days 10, 16, 26, and 47 DAS. The quantity of fertilizer was as follows: 30/15/30/34 kg N ha ^−1^, 30/0/30/34 kg P _2_O _5_ ha ^−1^, and 22.5/0/22.5/22.5 kg K _2_O ha ^−1^. Nitrogen (N), phosphorous (P), and potassium (K) were applied based on the application of urea, superphosphate, and potassium chloride fertilizer. Applied fertilizer quantities for field experiments are shown in the Table 2.

**Table 2.** Fertilizer quantities applied during the field experiment.

Fertilization	Applied fertilizer (kg ha ^−1^) ^†^	
	Wet season	Dry season
1 ^st^ topdressing	55, 25, 0	30, 30, 22.5
2 ^nd^ topdressing	42.5, 25, 0	15, 0, 0
3 ^rd^ topdressing	32, 25, 0	30, 30, 22.5
4 ^th^ topdressing	-	34, 34, 22.5

† Fertilizers applied were: N, P _2_O _5_, K _2_O (kg ha ^−1^).

### Measurements

Topsoil samples (10 cm) were collected by an auger with a 3.5 cm diameter. In the WS of 2018, we collected a soil sample before sowing to determine the soil's initial physicochemical properties. During the growth period, soil samples were taken on days 9, 13, 19, 27, 39, 53, 65, and 72 DAS. In the dry season of 2019, soil samples were collected on days 7, 14, 21, 29, 44, 52, 61, and 72 DAS. Samples were collected at five cross-sectional sites (four corners and one midpoint) and mixed to a similar weight to achieve a compromised sample. Fresh soil samples were removed of visible biomass, and air-dried and sieved at 2 mm. Soil texture was measured by sieving particle sizes to separate out coarse sand from the finer particles and the silt and clay contents were then determined by measuring the rate of settling of these two separates from the suspension in water according to the Robinson pipette method ( Carter and Gregorich, 2008). Bulk density samples were collected by core samplers and the cores were dried in an oven at 110 ^o^C until the weight was constant in accordance with the Core method ( Blake and Hartge, 1986). Soil organic matter (SOM) was oxidized by a K _2_Cr _2_O _7_-H _2_SO _4_ oxidation procedure and titrated using (NH _4_) _2_Fe(SO _4_) _2_(H _2_O) _6_ solution ( Walkley and Black, 1934). NH _4_^+^ was extracted by KCl 1M (1:10 soil/extract (wt:vol)) and measured according to the indophenol blue colorimetric method ( Lu, 2000). Total N (TKN) was digested in the digestion tablets (K _2_SO _4_, CuSO _4_, and Se) and H _2_SO _4_ solution at 375 ^o^C, then the digest was analyzed for NH _4_^+^ by the automated phenate method according to the Kjeldahl method ( Bremner, 1996). Total P (TP) was digested in sulphuric acid-hydrogen peroxide-hydrofluoric acid (H _2_SO _4_-H _2_O _2_-HF) and detected by the molybdenum blue method ( Bowman, 1988).

We also established a similar sampling program among SW, SbW, and soil. Likewise, SW samples were collected at soil sampling points and then mixed to obtain a joint representative sample. For SbW sampling, we installed five PVC pipes (120 cm in length and 9 cm in diameter) around the selected sampling points. The pipe was perforated by 2 mm holes and covered underneath by a lid. The perforated pipes were 15 cm in length. A plastic net of 2 mm was wrapped around the perforated pipe to avoid sediment intrusion. At each selected site, the pipe was anchored under the soil surface at 0.45 m depth. A lid was used to cover the pipe during non-sampling periods. NH _4_^+^, NO _3_^−^, PO _4_^3−^ and TP were analysed according to Standard Methods for the examination of Water and Wastewater (SMEWW) ( APHA, 1998): NH _4_^+^ was detected by the phenate method (SMEWW 4500-NH _3_ F), NO _3_^−^ was analysed by the automated hydrazine reduction method (SMEWW 4500-NO _3_^−^ G), PO _4_^3−^ was determined by the ascorbic acid method (SMEWW 4500-P E), TP was measured by the persulfate method for simultaneous determination of total phosphorus (SMEWW 4500-P J).

### Analysis

We assessed the nutrient variation in SW, SbW, and soil between the WS and DS and compared the concentration of water environmental parameters between SW and SbW. The differences between levels of each factor were analysed assuming equal variances (Student’s t-test) at a significant level of *P = *0.05 after passing the normality test (Shapiro-Wilk) ( *P* > 0.05). All computations were performed using R stats Version 4.2.0 (R Project for Statistical Computing, RRID:SCR_001905).

## Results

### N-NH _4_^+^, N-NO _3_^−^, P-PO _4_^3−^ and total phosphorus in the surface water and sub-surface water

**Figure 1.**
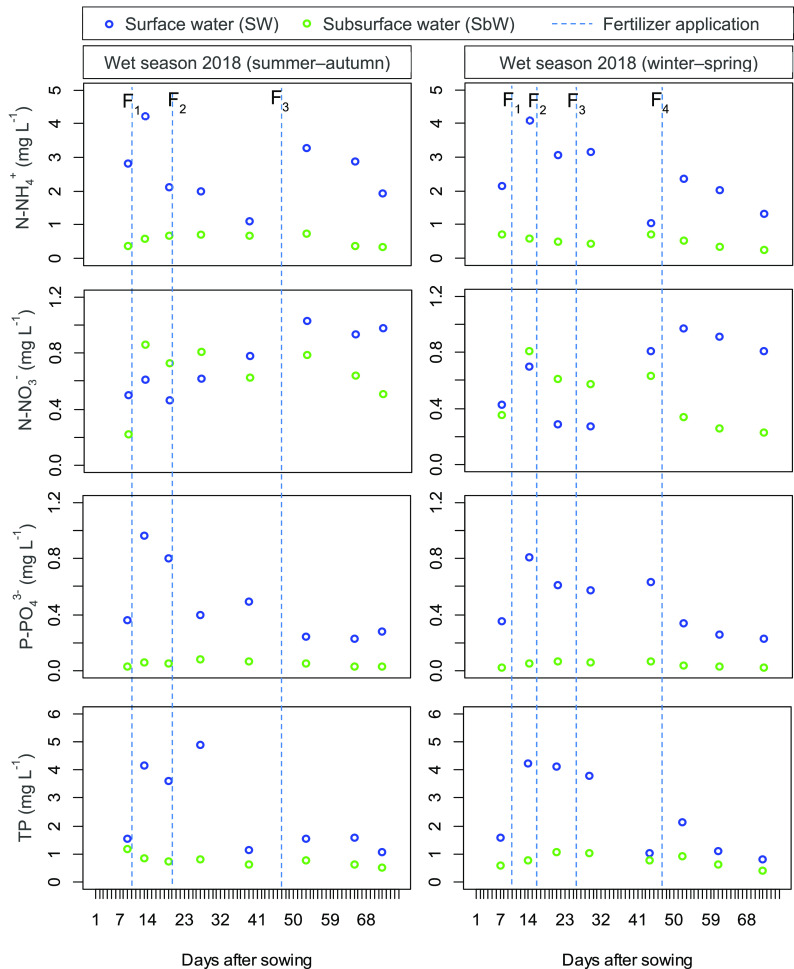
The variation of N-NH _4_^+^, N-NO _3_^−^, P-PO _4_^3−^ and total phosphorus in the surface water and sub-surface water during the wet season and dry season rice growing seasons. Vertical dotted lines indicate the times of the synthetic fertilizer application. F _1_, F _2_, F _3_ and F _4_ depict topdressing of fertilizer 1, 2, 3, and 4, respectively.

Nutrient variations in SW and SbW are shown in Figure 1. The concentrations of N-NH _4_^+^, N-NO _3_^−^_,_ P-PO _4_^3−^ and TP varied largely in the SW while remained relatively stable in the SbW. In particular, the nutrient values of the SW varied as follows: N-NH _4_^+^ (WS, 1.14–4.25 mg L ^−1^; DS, 1.03–4.09 mg L ^−1^), N-NO _3_^−^ (WS, 0.46–1.03 mg L ^−1^; DS, 0.27–0.97 mg L ^−1^) _,_ P-PO _4_^3−^ (WS, 0.23–0.96 mg L ^−1^; DS, 0.23–0.81 mg L ^−1^) _,_ and TP (WS, 1.06–4.89 mg L ^−1^; DS, 0.81–4.24 mg L ^−1^), while the concentration of nutrients in the SbW varied as follows: N-NH _4_^+^ (WS, 0.34–0.73 mg L ^−1^; DS, 0.24–0.71 mg L ^−1^), N-NO _3_^−^ (WS, 0.22–0.86 mg L ^−1^; DS, 0.23–0.81 mg L ^−1^) _,_ P-PO _4_^3−^ (WS, 0.03–0.08 mg L ^−1^; DS, 0.02–0.07 mg L ^−1^) _,_ and TP (WS, 0.51–1.19 mg L ^−^1; DS, 0.41–1.08 mg L ^−1^). The ratio of N-NH _4_^+^/N-NO _3_^−^ in the SW was consistently higher than in the SbW. Particularly, the rate of N-NH _4_^+^/N-NO _3_^−^ in the WS and DS varied by (SW, 1.4–6.9; SbW, 0.57–1.63) and (SW, 1.27–11.7; SbW, 0.72–2.0). It is noted that the concentration of N-NH _4_^+^, P-PO _4_^3−^ and TP in the SW was consistently higher than that in the SbW ( *P* < 0.05), while the value of N-NO _3_^−^ was insignificant between the SW and SbW ( *P* > 0.05). As observed, the highest peaks of these parameters were reached after fertilizing. It is likely that the higher concentration of N-NH _4_^+^, P-PO _4_^3−^ and TP in the SW were observed during the fertilizing period. NO _3_^−^ increased in the SbW during the fertilizing period, while the SW only rose in the WS and was more complex in the DS ( Figure 1). Although the temporal-spatial dynamics of the SW and SbW parameters were complex over the rice-growing period, statistical analysis indicates that for N-NH _4_^+^, N-NO _3_^−^, P-PO _4_^3−^ and TP; no significant differences between the WS and DS were seen ( Table 3).

**Table 3.** The average concentration of N-NH _4_^+^, N-NO _3_^−^_,_ P-PO _4_^3−^ and total phosphorus in surface water and sub-surface water and their interactions.

Factors	N-NH _4_^+^ (mg L ^−1^)	N-NO _3_^−^ (mg L ^−1^)	P-PO _4_^3−^ (mg L ^−1^)	TP (mg L ^−1^)
WS, *n = 8*				
SW	2.54 ± 0.96 **a**	0.74 ± 0.22	0.47 ± 0.27 **a**	2.44 ± 1.53 **a**
SbW	0.56 ± 0.17 **b**	0.65 ± 0.21	0.05 ± 0.02 **b**	0.77 ± 0.21 **b**
DS, *n = 8*				
SW	2.40 ± 1.01 **a**	0.65 ± 0.28	0.48 ± 0.21 **a**	2.35 ± 1.47 **a**
SbW	0.50 ± 0.17 **b**	0.48 ± 0.21	0.04 ± 0.02 **b**	0.78 ± 0.23 **b**
Two-seasonal variation (SV), *n = 16*				
SW	2.41 ± 0.95 **a**	0.69 ± 0.25	0.47 ± 0.23 **a**	2.40 ± 1.45 **a**
SbW	0.53 ± 0.17 **b**	0.51 ± 0.22	0.05 ± 0.00 **b**	0.78 ± 0.21 **b**
*P*-value				
WS (SW × SbW)	***	†	***	**
DS (SW × SbW)	***	†	***	**
SW (WS × DS)	†	†	†	†
GW (WS × DS)	†	†	†	†
SV (SW × SbW)	***	†	***	***

Means followed by different letters within each group of values indicate significance at *P* = 0.05 by the Student’s t-test.

WS, wet season; DS, dry season; SW, surface water; SbW, subsurface water; SV, two-seasonal variation. Data presented as means ± standard deviation ( *n* = 8) in the same column. Significant difference by t-test: *** *P* < 0.001, ** *P* < 0.01, * *P* < 0.05, and † *P* > 0.05.

### N-NH _4_^+^, total nitrogen, soil organic matter, and total phosphorus in the soil

**Figure 2.**
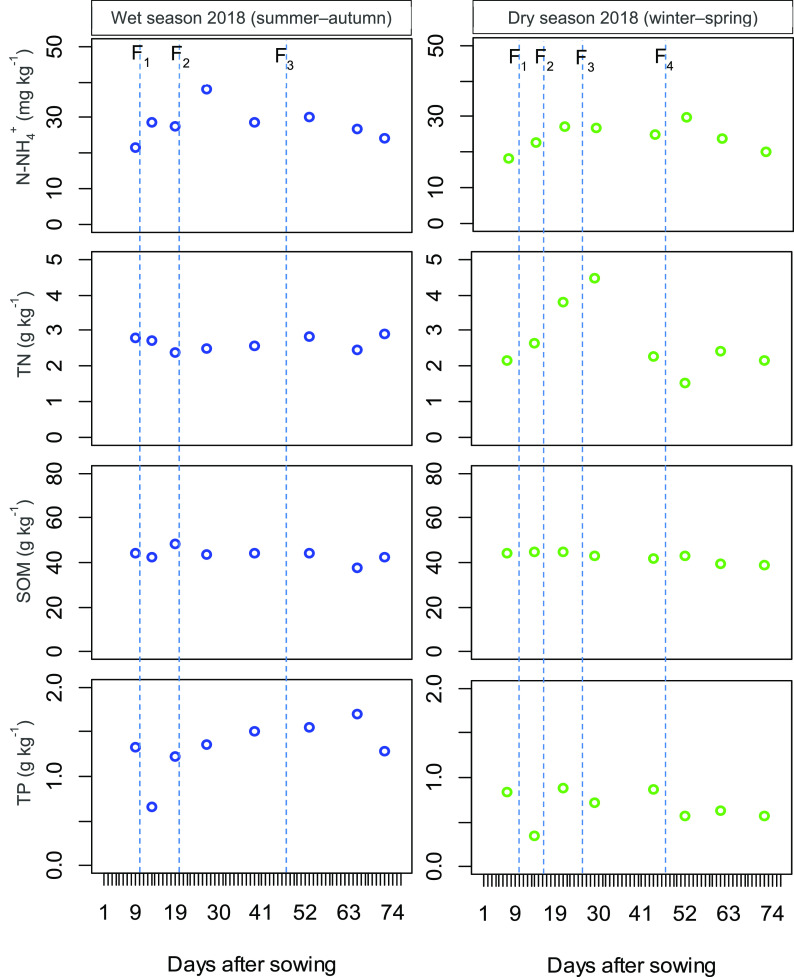
The variation of N-NH _4_^+^, total nitrogen, soil organic matter and total phosphorus in the soil during the rice growing wet season and dry season. Vertical dotted lines indicate the times of the synthetic fertilizer application. F _1_, F _2_, F _3_ and F _4_ depict topdressing of fertilizer 1, 2, 3, and 4, respectively. TP = total phosphorus; TN = total nitrogen.

Figure 2 shows the N-NH _4_^+^, TN, SOM, and TP variation in the soil paddy field over the WS and DS. In the WS, the concentration of soil chemical properties varied as follows: N-NH _4_^+^ (21.5–38.0 mg kg ^−1^), TN (2.37–2.90 g kg ^−1^), SOM (37.6–48.5 g kg ^−1^), and TP (0.65–1.69 g kg ^−1^), while the DS fluctuated as follows: N-NH _4_^+^ (18.41–29.6 mg kg ^−1^), TN (1.53–4.48 g kg ^−1^), SOM (38.7–44.9 g kg ^−1^), and TP (0.35–0.89 g kg ^−1^). The concentration of N-NH _4_^+^ increased relatively after fertilizer application in both the WS and DS. Likely, a similar trend was seen in the TN during the DS. However, the effects of synthetic fertilizer topdressing on the TN (in the WS), SOM, and TP were neglectable. Statistically, N-NH _4_^+^, TN, and SOM slightly increased in the WS ( *P* > 0.05), while TP significantly increased ( *P* < 0.001) ( Table 4).

**Table 4.** N-NH _4_^+^, total nitrogen, soil organic matter and total phosphorus in the wet season and dry season.

Factors	N-NH _4_^+^ (mg kg ^−1^)	TN (g kg ^−1^)	SOM (g kg ^−1^)	TP (g kg ^−1^)
WS	28.2 ± 4.83	2.64 ± 0.19	43.3 ± 3.02	1.32 ± 0.31 **a**
DS	24.2 ± 3.77	2.69 ± 0.97	42.4 ± 2.39	0.68 ± 0.19 **b**
*P*-value				
WS × DS	†	†	†	***

Means followed by different letters indicate significance at *P*=0.05 by the Student’s t-test.

WS, wet season; DS, dry season; TN, total nitrogen; TP, total phosphorus; SOM, soil organic matter. Data presented as means ± standard deviation ( *n* = 8) in the same column. Significant difference by t-test: *** *P* < 0.001, ** *P* < 0.01, * *P* < 0.05, and † *P* > 0.05.

## Discussion

### Variation of nutrient contents in the SW and SbW

Our study assessed nutrient variability in the SW and SbW through the WS and DS under typical water management and conventional rice practices in the Vietnamese MD. The study found that the concentration of N-NH _4_^+^, N-NO _3_^−^_,_ P-PO _4_^3−^, and total P in the SW exhibited a relatively large variation in the WS and DS ( Figure 1). This could be partly attributed to shifting water levels (rainfall and irrigation) and fertilizer application ( Qiao *et al.,* 2012). The alteration of SW levels could likely increase/decrease the denseness of constituents regarding the concentration/dilution in the rice field. In this study, we did not record the water levels as the farmer let water flow free on the paddy field. Thus, the interdependence between water levels and nutrient dynamics remains uncertain. However, fertilizer topdressings could also potentially stimulate the dynamic mineralization processes within the rice paddy field. Here, we found that higher N fertilizer applications (39.5 kg N ha ^−1^) in the WS slightly increased the average N-NH _4_^+^ and N-NO _3_^−^ concentration by 5.83% and 13.8%. In contrast, higher P fertilizer utilization (15 kg P _2_O _5_ ha ^−1^) in the DS was indistinguishable in cases of P-PO _4_^3−^, and TP ( Table 2). Thus, we suggest that an interaction between water levels and fertilizer application rates on the dynamics of N-NH _4_^+^, N-NO _3_^−^ , P-PO _4_^3−^, and TP in the Vietnamese MD’s paddy fields should be considered for further work.

Our study showed that the N-NH _4_^+^, N-NO _3_^−^ , P-PO _4_^3−^, and TP contents in the SW were consistently higher than that of the SbW simultaneously ( *P* < 0.001), irrespective of factors including fertilizer application rate and seasonal variation ( Ngan *et al.,* 2021). This means that nutrient loss mainly occurred through the SW. The lower nutrient concentrations in the SbW may also be partly explained by various transformation processes or plant uptake during percolation regression. For instance, the N-NH _4_^+^ could be reduced during leachate due to volatilization, nitrification (N-NH _4_^+^ to N-NO _3_^−^), and rice roots uptake ( Hou *et al.,* 2007). Furthermore, P-PO _4_^3−^ reduction could also be explained by rice plant uptake, binding onto soil minerals and being more prone to removal through surface runoff ( McDowell *et al.,* 2001). In line with our findings, Cho *et al.* (2011) found that leaching downward subsurface waters were responsible for 6.4–9.8% and 0.2–0.3% of N and P losses, respectively, while N and P losses via surface runoff accounted for 34.3–42.6% and 3.8–5.3%. It has been reported that nutrient loss during the rice-growing period is more relevant to the fertilizer application rate. Cui *et al.* (2020) confirmed that fertilizer applications significantly impacted N and P losses from surface runoff, with increased fertilizer application rates significantly increasing N loss through surface runoff. Besides, Qiao *et al.* (2012) showed that N loss via surface runoff and percolation positively correlated with fertilizer application rate. Also, the rate and timing of fertilizer application in the field influenced the N concentration loss over surface runoff ( Li *et al.,* 2018). These studies strongly supported our findings.

Our study found that N-NO _3_^−^ concentrations showed no significant difference between the SW and SbW, while N-NH _4_^+^ in the SbW was consistently lower than in the SW ( Ngan *et al.,* 2021). It is well-known that N-NH _4_^+^ in the rhizosphere area generally lowers the N-NO _3_^−^ as rice prefers N-NH _4_^+^, up taking N-NH _4_^+^ faster than N-NO _3_^−^. Moreover, nitrification progression also occurs very fast in the rhizosphere ( Li *et al.,* 2015; Li *et al.,* 2018). In the rice paddy field, N-NH _4_^+^ was simulated by both passive and active uptake, while N-NO _3_^−^ was solely simulated by a passive uptake ( Šimůnek and Hopmans, 2009). This meant that rice uptakes more N-NH _4_^+^ than N-NO _3_^−^. In line with these findings, Kirk and Kronzucker (2005) depicted that rice could absorb N-NH _4_^+^ from 60% to 85%, while 85% of N leaching loss exists in the N-NO _3_^−^ form; a higher concentration of N-NO _3_^−^ found below the soil surface (mostly in the rhizosphere) resulted in it being quickly transported to the soil surface ( Mo’allim *et al.,* 2018). As such, it is evident that N-NO _3_^−^ tends to be high in SbW, which is consistent with our findings. However, it is noted that the change between N-NH _4_^+^ and N-NO _3_^−^ pertains to rainfall, surface runoff, and irrigation ( Li *et al.,* 2015). Thus, we propose that the underlying nutrient dynamics in rice paddy fields under different water management regimes and differences of rice farming practices in the lowland soils of the Vietnamese MD should be further studied in future work.

### Variation of soil nutrients in rice paddy fields

This study described the soil chemical properties during the rice-growing period under conventional rice practices in the Vietnamese MD. Variability of N-NH _4_^+^, TN, SOM, and TP during rice growth was comparable to the previous studies undertaken in the lowland soils of the MD ( Minamikawa *et al.*, 2021; Vo *et al.*, 2018; Uno *et al.*, 2021). According to Hung *et al.* (2016) and Tanaka *et al.* (2014), soil properties in our study were characterized by medium-high TN, high N-NH _4_^+^, low-to-medium SOM, and high-to-very high TP. Thus, with respect to ensuring soil responsiveness to rice nutrient demand, reducing the N and P fertilizer application rate, and increasing SOM to a feasible degree should be considered in conventional rice practices in the Vietnamese MD.

We found that soil N-NH _4_^+^ and TN concentration slightly increased after fertilizing. Higher N-fertilizer application (39.5 kg N ha ^−1^) in the WS insignificantly increased the N-NH _4_^+^ and TN in the soil in comparison to that of the DS ( *P* > 0.05). However, higher fertilizer application of 15 kg P _2_O _5_ ha ^−1^ significantly increased TP in the soil paddy field ( *P* < 0.001). It is indicated that the fertilizer application moderately boosted the dynamic of N availability in soil. In agreement with our study, Dong *et al.* (2012) confirmed that available N slightly increased with chemical fertilizer application but significantly increased with organic matter additions. It is noted that the significant difference in TP could be likely due to the excessive P fertilizer application rates in the WS, while utilization efficacy and loss of P are usually low ( Irfan *et al.,* 2020; Schröder *et al.,* 2011; Cho *et al.,* 2011).

SOM plays an inevitable role in promoting nutrient availability and improving soil fertility. Our study found that SOM change was minor during rice growth. This implied that the regression of organic matter mineralization/decomposition could occur slowly. Moreover, no organic matter was added to the soil in the paddy field. Thus, organic matter ineffectively contributed to nutrient availability in the soil. In the soil, change of SOM depends on temperature, pH, microbial growth, soil management, organic matter amendment, and C/N ratio ( Tanaka *et al.,* 2014).

## Conclusions

This study examined the temporal-spatial variability of nutrients in SW, SbW, and soil of a paddy field in the WS and DS under typical water management and conventional cultivation techniques. We found that nutrient content in the SW showed a high fluctuation during the rice-growing period, while stability was observable in the SbW. After fertilizer application, the highest peaks of N-NH _4_^+^, P-PO _4_^3−^ and TP parameters in the SW and SbW were observed. The concentrations of N-NH _4_^+^, P-PO _4_^3-,^ and TP in the SW were all-time higher than that of the SbW. While N-NO _3_^−^ concentration was insignificant between the SW and SbW. The seasonal nutrient variations were insignificant in both the SW and SbW. Our findings showed that soil properties were characterized by medium-high TN, high N-NH _4_^+^, low-to-medium SOM, and high-to-very high TP. Higher N fertilizer application slightly increased the N-NH _4_^+^ and TN dynamic, while TP significantly increased along with increasing P fertilizer application rate in the WS. SOM showed stability during both the WS and DS. We suggest that nutrient loss estimations and dynamic simulations in the lowland soil of the Vietnamese MD’s rice paddy fields should be considered for further work.

## Data availability

### Underlying data

Figshare: Underlying data for ‘Nutrient dynamics in water and soil under conventional rice cultivation in the Vietnamese Mekong Delta’. ‘Paddy field in the Vietnamese Mekong Delta 2021’, https://doi.org/10.6084/m9.figshare.16499508.v1 ( Ngan *et al*., 2021).

This project contains the following underlying data:

• Supplementary file – 2021.xlsx

Data are available under the terms of the Creative Commons Zero “No rights reserved” data waiver (CC0 1.0 Public domain dedication).

## References

[ref1] APHA: *Standard methods for the examination of water and wastewater.* Washington DC, USA: American Public Health Association, American Water Works Association and Water Environmental Federation; 20th ed. 1998.

[ref2] Blake GR Hartge KH : Bulk density and particle density. *‘Methods of soil analysis. Part 1’. Agronomy Monograph 9.* Klute A , editor. Madison, WI: ASA and SSSA;1986; pp.363–382.

[ref3] Bowman RA : A rapid method to determine total phosphorus in soils. *Soil Sci. Soc. Am. J.* 1988;52:1301–1304. 10.2136/sssaj1988.03615995005200050016x

[ref4] Bremner JM : Nitrogen-total. *Methods of soil analysis: Part 3 Chemical methods, 5.3; SSSA Book Series.* Sparks D Page A Helmke P Loeppert R , editors. Madison, WI, USA: Wiley & Sons;1996; pp.1085–1121.

[ref5] Carter MR Gregorich EG : Soil sampling and methods of analysis. *Canadian Society of Soil Science.* 2008;1240.

[ref6] Cho J-Y Han K-W Choi J-K : Nutrient losses from a paddy field plot in Central Korea. *Water Air Soil Pollut.* 2011;48:301–306. 10.1080/00380768.2002.10409205

[ref7] Clauss K Ottinger M Leinenkugel P : Estimating rice production in the Mekong Delta, Vietnam - Utilizing time series of Sentinel-1 SAR data. *Int. J. Appl. Earth Obs. Geoinf.* 2018;73:574–585. 10.1016/j.jag.2018.07.022

[ref8] Connor M Tuan LA DeGuia AH : Sustainable rice production in the Mekong River Delta: Factors influencing farmers’ adoption of the integrated technology package “One must do, five reductions” (1M5R). *Outlook Agric.* 2021;50:90–104. 10.1177/0030727020960165

[ref9] Cui N Cai M Zhang X : Runoff loss of nitrogen and phosphorus from a rice paddy field in the East of China: Effects of long-term chemical N-fertilizer and organic manure applications. *Glob. Ecol. Conserv.* 2020;22:e01011. 10.1016/j.gecco.2020.e01011

[ref10] DONRE: *Environment status report from 2015 – 2019 in Can Tho city.* Can Tho city, Vietnam: Department of Natural Resources and Environment in Can Tho city;2020.

[ref11] Dong W Zhang X Wang H : Effect of different fertilizer application on the soil fertility of paddy soils in red soil region of Southern China. *PLoS One.* 2012;7:e44504. , 10.1371/journal.pone.0044504 23028550 PMC3460972

[ref12] Hou H Zhou S Hosomi M : Ammonia emissions from anaerobically-digested slurry and chemical fertilizer applied to flooded forage rice. *Water Air Soil Pollut.* 2007;183:37–48. 10.1007/s11270-007-9353-9

[ref13] Hung NN Ve NB Minh VQ : *Management of soil fertility in the Mekong Delta, Vietnam.* Can Tho city: Can Tho University publisher, Vietnam;2016;513. (in Vietnamese)

[ref14] Irfan M Aziz T Maqsood MA : Phosphorus (P) use efficiency in rice is linked to tissue-specific biomass and P allocation patterns. *Sci. Rep.* 2020;10:4278. 10.1038/s41598-020-61147-3 32152340 PMC7062884

[ref15] International Union of Soil Sciences (IUSS) Working Group World Reference Base (WRB): *World Reference Base for Soil Resources 2014, updated 2015. International soil classification system for naming soils and creating legends for soil maps. World Soil Resources Reports No. 106.* Rome, Italy: FAO;2015.

[ref16] Kirk GJD Kronzucker HJ : The potential for nitrification and nitrate uptake in the rhizosphere of wetland plants: A modelling study. *Ann. Bot.* 2005;96:639–646. 10.1093/aob/mci216 16024557 PMC4247031

[ref17] Li P Lu J Wang Y : Nitrogen losses, use efficiency, and productivity of early rice under controlled-release urea. *Agric. Ecosyst. Environ.* 2018;251:78–87. 10.1016/j.agee.2017.09.020

[ref18] Li Y Šimůnek J Zhang Z : Evaluation of nitrogen balance in a direct-seeded-rice field experiment using Hydrus-1D. *Agric. Water Manag.* 2015;148:213–222. 10.1016/j.agwat.2014.10.010

[ref19] Lu RK : *Methods of soil and agro-chemical analysis.* Beijing, China: China Agricultural Science and Technology Press;2000.

[ref20] McDowell RW Sharpley AN Condron LM : Processes controlling soil phosphorus release to runoff and implications for agricultural management. *Nutr. Cycl. Agroecosyst.* 2001;59:269–284. 10.1023/A:1014419206761

[ref21] Minamikawa K Huynh KC Uno K : Cattle biogas effluent application with multiple drainage mitigates methane and nitrous oxide emissions from a lowland rice paddy in the Mekong Delta, Vietnam. *Agric. Ecosyst. Environ.* 2021;319:107568. 10.1016/j.agee.2021.107568

[ref22] Mo’allim A Kamal M Muhammed H : Assessment of nutrient leaching in flooded paddy rice field experiment using Hydrus-1D. *Water.* 2018;10:785. 10.3390/w10060785

[ref23] Ngan N-V-C Huynh Van T Giang Nam N-D : Paddy field in the Vietnamese Mekong Delta 2021. Figshare. *Dataset.* 2021. 10.6084/m9.figshare.16499508.v1

[ref24] Peng S-Z Yang S-H Xu J-Z : Nitrogen and phosphorus leaching losses from paddy fields with different water and nitrogen managements. *Paddy Water Environ.* 2011;9:333–342. 10.1007/s10333-010-0246-y

[ref25] Qi D Wu Q Zhu J : Nitrogen and phosphorus losses from paddy fields and the yield of rice with different water and nitrogen management practices. *Sci. Rep.* 2020;10:9734. 10.1038/s41598-020-66757-5 32546803 PMC7297741

[ref26] Qiao J Yang L Yan T : Nitrogen fertilizer reduction in rice production for two consecutive years in the Taihu lake area. *Agric. Ecosyst. Environ.* 2012;146:103–112. 10.1016/j.agee.2011.10.014

[ref27] Scalenghe R Edwards AC Barberis E : Release of phosphorus under reducing and simulated open drainage conditions from over fertilised soils. *Chemosphere.* 2014;95,289–294. 10.1016/j.chemosphere.2013.09.016 24103442

[ref28] Schröder JJ Smit AL Cordell D : Improved phosphorus use efficiency in agriculture: A key requirement for its sustainable use. *Chemosphere.* 2011;84:822–831. 10.1016/j.chemosphere.2011.01.065 21349568

[ref29] Shankar T Banerjee M Malik GC : The productivity and nutrient use efficiency of rice–rice–black gram cropping sequence are influenced by location specific nutrient management. *Sustainability.* 2021;13:3222. 10.3390/su13063222

[ref30] Šimůnek J Hopmans JW : Modeling compensated root water and nutrient uptake. *Ecol. Model.* 2009;220:505–521. 10.1016/j.ecolmodel.2008.11.004

[ref31] Stuart AM Devkota KP Sato T : On-farm assessment of different rice crop management practices in the Mekong Delta, Vietnam Using Sustainability Performance Indicators. *Field Crop Res.* 2018;229:103–114. 10.1016/j.fcr.2018.10.001

[ref32] Tanaka H Katsuta A Toyota K : Soil fertility and soil microorganisms. *Research approaches to sustainable biomass systems.* Elsevier;2014; pp.107–142. 10.1016/B978-0-12-404609-2.00005-2

[ref33] Uno K Ishido K Nguyen Xuan L : Multiple drainage can deliver higher rice yield and lower methane emission in paddy fields in An Giang Province, Vietnam. *Paddy Water Environ.* 2021;19:623–634. 10.1007/s10333-021-00861-8

[ref34] Vo TBT Wassmann R Tirol-Padre A : Methane emission from rice cultivation in different agro-ecological zones of the Mekong River Delta: Seasonal patterns and emission factors for baseline water management. *Soil Sci. Plant Nutr.* 2018;64:47–58. 10.1080/00380768.2017.1413926

[ref35] Walkley A Black IA : An Examination of the Degtjareff method for determining organic carbon in soils: Effect of variations in digestion conditions and of inorganic soil constituents. *Soil Sci.* 1934;63:251–264. 10.1097/00010694-194704000-00001

[ref36] Wang L Huang D : Nitrogen and phosphorus losses by surface runoff and soil microbial communities in a paddy field with different irrigation and fertilization managements. *PLoS One.* 2021;16:e0254227. 10.1371/journal.pone.0254227 34242302 PMC8274659

[ref37] Wang Y Zhao X Wang L : The regime and P availability of omitting P fertilizer application for rice in rice/wheat rotation in the Taihu lake region of Southern China. *J. Soils Sediments.* 2015;15:844–853. 10.1007/s11368-014-1047-5

[ref38] Yang S Peng S Xu J : Effects of water saving irrigation and controlled release nitrogen fertilizer managements on nitrogen losses from paddy fields. *Paddy Water Environ.* 2015;13:71–80. 10.1007/s10333-013-0408-9

[ref39] Zhu ZL Chen DL : Nitrogen fertilizer use in China - Contributions to food production, impacts on the environment and best management strategies. *Nutr. Cycl. Agroecosyst.* 2002;63:117–127. 10.1023/A:1021107026067

