# Supplementary material for: Nutrient dynamics in water and soil under conventional rice cultivation in the Vietnamese Mekong Delta
Source: F1000Res. 2023 Jan 5;10:1145. Originally published 2021 Nov 11. [Version 2] doi: 10.12688/f1000research.73904.2 (PMC10835106; doi:10.12688/f1000research.73904.2)
Supplement: Peer review history

# Reviewer report: Reviewer response for version 2

**DOI:** 10.5256/f1000research.141379.r235731

**Published:** 2024-02-01

**Authors:** Rahul Adhikary

**Affiliations:** 1 M.S. Swaminathan School of Agriculture and Centurion University of Technology and Management, Odisha, India

Novelty of the proposed work should be established by comparing the same with comparable work. 

 citation established the credibility of a research work but have been poorly used.

 Literature review is shallow and future scope is missing.

 At discussion part some citation are more than 10 years old, it should be recent.

Is the work clearly and accurately presented and does it cite the current literature?

Partly

If applicable, is the statistical analysis and its interpretation appropriate?

I cannot comment. A qualified statistician is required.

Are all the source data underlying the results available to ensure full reproducibility?

Partly

Is the study design appropriate and is the work technically sound?

Yes

Are the conclusions drawn adequately supported by the results?

Partly

Are sufficient details of methods and analysis provided to allow replication by others?

Partly

Reviewer Expertise:

Fertility management, problem soil management

I confirm that I have read this submission and believe that I have an appropriate level of expertise to confirm that it is of an acceptable scientific standard, however I have significant reservations, as outlined above.

# Reviewer report: Reviewer response for version 2

**DOI:** 10.5256/f1000research.141379.r223581

**Published:** 2024-01-29

**Authors:** Arnab Majumdar

**Affiliations:** 1 School of Environmental Studie, Jadavpur University, Kolkata, West Bengal, India; 2 Department of Earth Sciences, Indian Institute of Science Education and Research Kolkata, Mohanpur, West Bengal, India

The article is considerable with the data set presented here; however, some suggestions are given which should be followed for a better representation-

 1. How the differential water content is affecting soil physico-chemical parameters should be discussed more clearly. Please refer to this below-given article-

 Majumdar, A., Upadhyay, M.K., Giri, B., Srivastava, S., Srivastava, A.K., Jaiswal, M.K. and Bose, S., 2021. Arsenic dynamics and flux assessment under drying-wetting irrigation and enhanced microbial diversity in paddy soils: A four year study in Bengal delta plain. *Journal of Hazardous Materials*, *409*, p.124443.

 2. The distribution of soil nutrients under differential water content and rice plant associations could have been discussed more thoroughly. Please refer to the below-given article-

 Majumdar, A., Dubey, P.K., Giri, B., Moulick, D., Srivastava, A.K., Roychowdhury, T., Bose, S. and Jaiswal, M.K., 2023. Combined effects of dry-wet irrigation, redox changes and microbial diversity on soil nutrient bioavailability in the rice field. *Soil and Tillage Research*, *232*, p.105752.

 3. What might be the bioconcentration factor of these nutrients translocating from soil to rice plants ?

Is the work clearly and accurately presented and does it cite the current literature?

Partly

If applicable, is the statistical analysis and its interpretation appropriate?

Partly

Are all the source data underlying the results available to ensure full reproducibility?

Yes

Is the study design appropriate and is the work technically sound?

Yes

Are the conclusions drawn adequately supported by the results?

Partly

Are sufficient details of methods and analysis provided to allow replication by others?

Yes

Reviewer Expertise:

Soil-Plant Biogeochemistry and Environmental Microbiology

I confirm that I have read this submission and believe that I have an appropriate level of expertise to confirm that it is of an acceptable scientific standard, however I have significant reservations, as outlined above.

## References

[1] : Arsenic dynamics and flux assessment under drying-wetting irrigation and enhanced microbial diversity in paddy soils: A four year study in Bengal delta plain. *J Hazard Mater* .2021;409: 10.1016/j.jhazmat.2020.124443 124443 10.1016/j.jhazmat.2020.124443 33191021

[2] : Combined effects of dry-wet irrigation, redox changes and microbial diversity on soil nutrient bioavailability in the rice field. *Soil and Tillage Research* .2023;232: 10.1016/j.still.2023.105752 10.1016/j.still.2023.105752

# Reviewer report: Reviewer response for version 2

**DOI:** 10.5256/f1000research.141379.r159250

**Published:** 2023-01-24

**Authors:** Koki Toyoda

**Affiliations:** 1 " Graduate School of Bio-Applications and Systems Engineering", Tokyo University of Agriculture and Technology, Tokyo, Japan

I have just one comment.

 P>0.05

 In case that a P value is more than 0.05, the actual value, like P=0.20, should be written.

 I have no other comments.

Is the work clearly and accurately presented and does it cite the current literature?

Yes

If applicable, is the statistical analysis and its interpretation appropriate?

Yes

Are all the source data underlying the results available to ensure full reproducibility?

Yes

Is the study design appropriate and is the work technically sound?

Yes

Are the conclusions drawn adequately supported by the results?

Yes

Are sufficient details of methods and analysis provided to allow replication by others?

Yes

Reviewer Expertise:

NA

I confirm that I have read this submission and believe that I have an appropriate level of expertise to confirm that it is of an acceptable scientific standard.

# Reviewer report: Reviewer response for version 1

**DOI:** 10.5256/f1000research.77593.r135271

**Published:** 2022-05-04

**Authors:** Koki Toyoda

**Affiliations:** 1 " Graduate School of Bio-Applications and Systems Engineering", Tokyo University of Agriculture and Technology, Tokyo, Japan

This manuscript reports valuable field data regarding nutrient dynamics in a paddy field. The results of differences between two depths and two seasons are interesting and worth to be published. However, there are several parts that are poorly explained or lack clearness. I commented directly on the manuscript. These comments should be properly amended.

 The annotated manuscript can be found at the following link: *https://f1000researchdata.s3.amazonaws.com/linked/419959.Koki_Toyota_review_ngan_nguyen-vo-chau.pdf*

Is the work clearly and accurately presented and does it cite the current literature?

Yes

If applicable, is the statistical analysis and its interpretation appropriate?

Yes

Are all the source data underlying the results available to ensure full reproducibility?

Yes

Is the study design appropriate and is the work technically sound?

Yes

Are the conclusions drawn adequately supported by the results?

Yes

Are sufficient details of methods and analysis provided to allow replication by others?

Yes

Reviewer Expertise:

NA

I confirm that I have read this submission and believe that I have an appropriate level of expertise to confirm that it is of an acceptable scientific standard, however I have significant reservations, as outlined above.

# Response

**Published:** 2022-11-21

**Authors:** Ngan Nguyen-Vo-Chau

**Affiliations:** Can Tho University, Vietnam

**Response to reviewer:**

 Thank you very much for giving us constructive comments that helped us improve this paper immensely. All your comments have been carefully read and revised to elucidate underlying aspects. Our responses to your comments are as follows:

- **Abstract:**

**1.1 Comment 1 & comment 2**: please describe the contents.  I guess the following expression is preferable. "Surface water, subsurface water, and topsoil samples were collected eight times during the ***"; during the rice-growing seasons to estimate/clarify ?????

**Answer:** Thank you so much for your commendation. The recommendation has been adopted. We revised it as follows “ ***Surface water, subsurface water (30–45 cm), and topsoil (0–20 cm) were collected eight times during the rice-growing seasons to clarify its nutrient dynamic”***.

**1.2 Comment 2:** describe the P-value in case of P>0.05.

**Answer:** We rephrased the sentence as follows: ***“*** ***no significant difference was disclosed (P>0.05)” *** to make it clearer to readers. Detailed interpretation of the p-value was cleared in the methodology section “A P-value less than 0.05 was statistically significant, while a P-value greater than 0.05 indicated no effect. Significantly different comparison was considered at ***P < 0.001, **P < 0.01, *P < 0.05 and †P > 0.05.”

**1.3 Comment 3:** “total P losses mainly occur through runoff rather than leaching” à better to describe the results supporting this conclusion.

**Answer:** We added the result to support the conclusion. It is as follows ***“*** ***Under conventional rice cultivation, the low concentration of N-NH _4_^+^, P-PO _4_^3-^ and total P in the subsurface water indicated that nutrient losses mainly occur through runoff rather than leaching”.***

**1.4 comment 4: *“*** While N-NO _3_^-^ loss is similar in surface water and subsurface water” à not clear. How do the authors define "loss"?

**Answer:** ***We agree with the reviewer that the conclusion is not clear and did not strongly support the conclusion. It is, therefore, eliminated.***

**1.5 Comment 5: **“dynamic simulation” à not clear

**Answer:** We revised the sentences as follows: ***“*** ***Future work should consider the nutrient balance and nutrient dynamic simulation in surface and subsurface of the lowland paddy soil in the Vietnamese Mekong Delta”.***

- **Introduction:**

**2.1 Comment 1:** “common practice” à common practice in the MD or VN

**Answer:** It has been corrected in the context of the mentioned paper. The revision is as follows: **“Common practices in the MD typically use fertilizers, water, and seeds well exceeding recommended rates”.**

**2.2 Comment 2:** “recommend rate” by ??

**Answer:** We added more information to make it clear to readers. The supplementary information is as follows: “Common practices in the MD typically use fertilizers, water, and seeds well exceeding recommended rates sustainable rice farming practices combined in the national program “One Must Do, Five Reductions” (1M5R)”.

**2.3 Comment 3:** “Nutrient migration was lost” à not clear

**Answer:** We revised this sentence to make it clearer to readers which is as follows: ***Several previous studies reported that nutrient losses primarily occurred via surface water (SW) and sub-surface water (SbW)***.

- **Methods:**

**3.1 Comment 1 “**sand, 1.9%, and clay, 66.4%” à silt ??%.

**Answer:** We supplemented the percentage of silt as follows: **“soil texture (sand, 1.9%, silt, 31.7%, and clay, 66.4%)”.**

**3.2 Comment 2 “**soil organic matter (SOM), 35.4 mg kg ^−1^” à too low value

**Answer:** Thank you so much for recognizing the low value. This was our miswriting when it came to filling the unit. It has been adopted and revised as follows: ***“soil organic matter (SOM), 35.4 g kg ^−1^*** ***“***

- **Results:**

**4.1 Comment 1: **“P > 0.05” à describe the P value

**Answer:** Please see our response in ***“comment 1.2”***

**4.2 Comment 2: N-NH _4_^+^ (18.41–29.6 mg kg ^−1^)** **à** **18.4**

**Answer:** It has been adopted. Revision is as follows: **“N-NH _4_^+^ (18.4–29.6 mg kg ^−1^)”**

- **Discussion:**

**4.1 Comment 1:** “This means that nutrient loss mainly occurred through the SW” à This is a bit radical interpretation. Please explain more logically why the authors describe "mainly". In addition to this hypothesis, uptake of the nutrients by rice plants must also occur.

**Answer:** Thank you so much for providing a hypothesis related to rice plant absorption. Actually, our hypothesis was impressing the loss of nutrients via the SW pathway rather than SbW percolation. In the rice ecosystems, the loss could be attributed to runoff, evaporation (nitrogen gases), and rice plant absorption. We seriously adopted your mentioned hypothesis to amend the explanation. The modification is as follows **“This means that nutrient losses could occur through the SW runoff, evaporation (nitrogen gases) and rice plant absorption rather than leaching to SbW”**

**4.2 Comment 2: “**N concentration loss” à **??**

**Answer:** We removed “concentration” has been eliminated. It is as follows **“N loss”**

**4.3 Comment 3:** “ It is well-known that N-NH _4_^+^ in the rhizosphere area generally lowers the N-NO _3_^-^ as rice prefers N-NH _4_^+^, up taking N-NH _4_^+^ faster than N-NO _3_^-^ “ à not clear

**Answer:** We rephrased the sentence in cohesion with the first sentence which is as follows: “ Our study found that N-NO _3_^-^ concentrations showed no significant difference between the SW and SbW, while N-NH _4_^+^ in the SbW was consistently lower than in the SW (Ngan et al., 2021). ***This could be partly explained by rice plants preferring uptake of N-NH _4_^+^ rather than N-NO _3_^-^, resulting in higher N-NO _3_^-^ concentration disclosed in the SbW***”.

**4.4. Comment 4:** “a higher concentration of N-NO _3_^−^ found below the soil surface (mostly in the rhizosphere) resulted in it being quickly transported to the soil surface”**** *à* *correct? to the subsoil?*

**Answer:** Thank you for correcting the expression. It has been adopted. Revision is as follows “a higher concentration of N-NO _3_^−^ found below the soil surface (mostly in the rhizosphere) resulted in it being quickly transported to the subsoil”.

**4.5 Comment 5:** This implied that the regression of organic matter mineralization/decomposition could occur slowly à not clear

**Answer:** We agree with the reviewer that the expression did not clear. Thus, we simplified this sentence as follows: This implied that the regression of organic matter mineralization/decomposition could occur slowly “SOM plays an inevitable role in promoting nutrient availability and improving soil fertility. Our study found that SOM change was minor during rice growth. This could be partly explained by no organic matter sources being incorporated into the soil.”

 We look forward to hearing from you.

 Best regards,

 Authors.
